# Supplementary material for: Screening and Identification of Key Biomarkers in Metastatic Uveal Melanoma: Evidence from a Bioinformatic Analysis
Source: J Clin Med. 2022 Dec 5;11(23):7224. doi: 10.3390/jcm11237224 (PMC9739237; doi:10.3390/jcm11237224)
Supplement: Supplementary file 1 [file jcm-11-07224-s001.zip › jcm-2017830-supplementary.pdf]

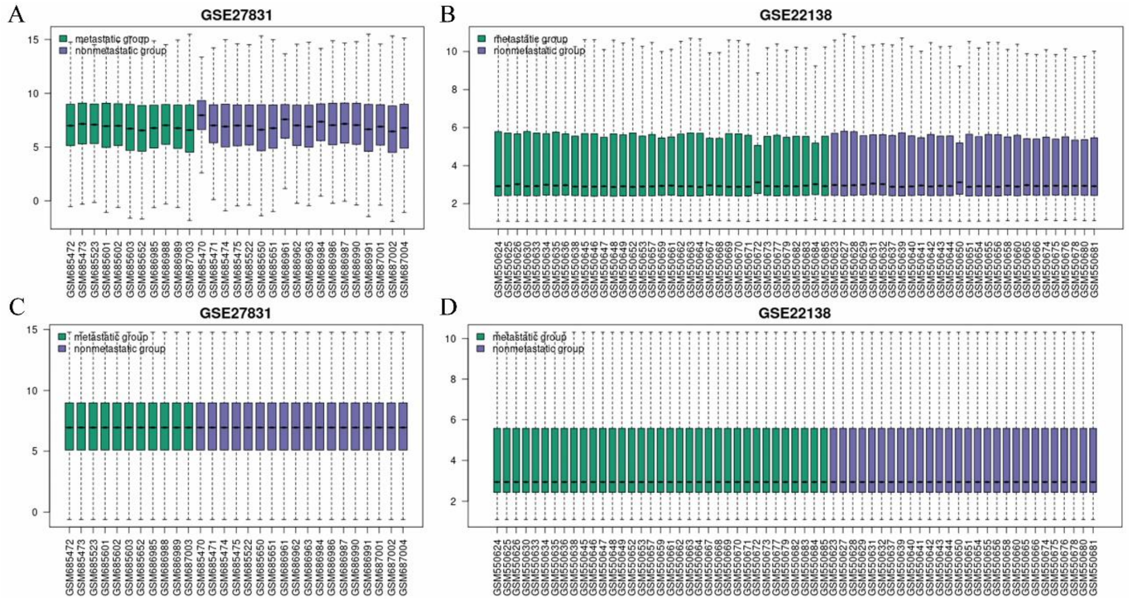

Figure S1. Box plots of the gene expression data before and after normalization. (**A**) The data before standardization of GSE27831, (**B**) the data before standardization of GSE22138, (**C**) the data after standardization of GSE27831, (**D**) the data after standardization of GSE22138. The x-axis label represents the sample symbol, and the y-axis label represents the gene expression values. The black line in the box plot represents the median value of gene expression. The green bar represents the metastatic group, and the purple bar represents the nonmetastatic group.

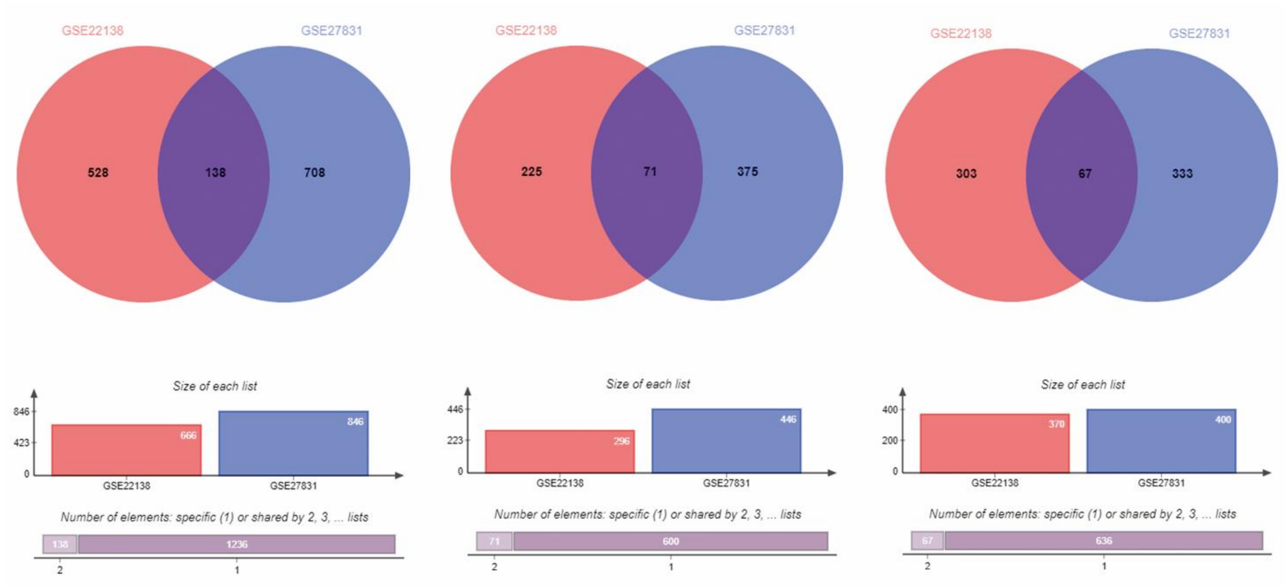

(**A**) (**B**) (**C**)

Figure S2. Venn diagram of common differentially expressed genes from the two datasets. (**A**) Coexpressed DEGs in the two datasets. (**B**) Seventy-one DEGs were downregulated in the two datasets. (**C**) Sixty-seven DEGs were upregulated in the two datasets.

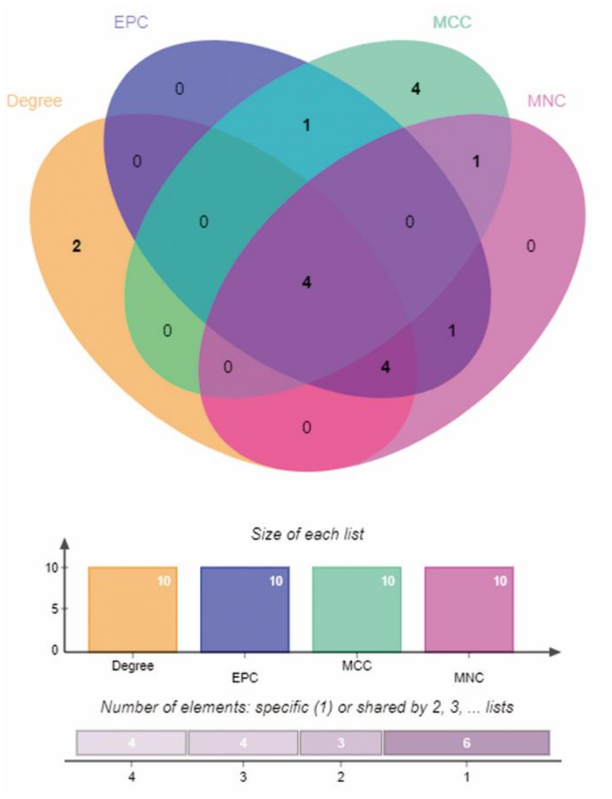

Figure S3. Venn diagram of the degree, edge percolated component, maximal clique centrality algorithm, and maximum neighborhood component algorithm scores of DEGs calculated through the CytoHubba plugin.
